# New Hampshire Veterinary Medical Association

**Published:** 1901-03

**Authors:** Lemuel Pope

**Affiliations:** Secretary


Secretary.

### NEW HAMPSHIRE VETERINARY MEDICAL ASSOCIATION.

THE old Association was reorganized and incorporated February 20, 1901. The first meeting was held in Manchester, with Drs. Bailey, Ebbett, Clark, Dodge, Dunton, Macguire, Hart, Burchsted, Bodwell, Byne, Russell, Loring, Wadsworth, and Pope present. The following officers were elected for the ensuing year: Dr. G. E. Chesley, President; Dr. C. E. Bailey, Vice-President; Dr. L. Pope, Secretary and Treasurer. Drs. Bodwell, Loring, and Burchsted, Executive Committee.

The old constitution was adopted with but few changes. Dr. Pope read a paper on "Azoturia" and a general discussion followed. A previous step taken toward legislation necessitated considerable discussion at this time and a committee was appointed to act in connection with our attorney.

Adjourned until the first Friday in April; meeting to be held in Manchester. LEMUEL POPE, JR., M.D.V.,
Secretary.

